# The contribution of a hermeneutic approach to investigate psychological variables in second language acquisition

**DOI:** 10.3389/fpsyg.2022.1055249

**Published:** 2022-12-02

**Authors:** Qing Chang

**Affiliations:** School of Foreign Languages, Anshan Normal University, Anshan, China

**Keywords:** hermeneutics, modern social hermeneutic, psychological variables, second language acquisition, qualitative research, quantitative methods

## Abstract

Qualitative research and more specifically a hermeneutic approach to interpreting communications in L2 classes has much to contribute to the accumulation of psychological knowledge about L2 learners. The modern social hermeneutic approach helps to address questions that are quite relevant for explaining contemporary developments in L2 educational strategies and policy-making. It can be used alone or in combination with quantitative methods of data analysis to analyze how certain psychological variables (cognitive factors, affective factors, and personality traits) emerge from the social interactions that occur within the ecologically situated nature of classroom language learning. The purpose of this conceptual review is to provide an introductory overview of the hermeneutic approach and discuss how it can be effectively used in L2 studies to explore the emergence, development, and causal mechanisms underlying the psychological variables in the interactive context of L2 classes. Also, suggestions will be explained with respect to the contributions of the method to L2 psychology, educational psychology, and second language acquisition domains.

## Introduction

On broad terms, hermeneutics is defined as the art and science of interpreting texts, which has been around for problem solving since Plato and Aristotle from old Greece (*cf.* Grondin, 1994; Hufnagel, 2000; Joisten, 2009). The roots of the term lie within the Greek word *hermeneuein*, with different meanings including ‘to explain’, ‘to describe, ‘to translate and ‘to clarify, and it entails different ways of interpreting written content, especially texts related to the religion, law, and literature (Lueger and Vettori, 2014). The roots of the hermeneutic approach can be also traced back to Schütz’ phenomenological theory, which points to the already-existing nature of perceptions in the form of integrative structures of meaning (Schütz, 1972, 1982). Viewed this way, the current experiences are hardly separable from prior experiences. These experiences are mostly gained and formed within human communication and interactions with other individuals and are added to the socially shared stocks of knowledge and experience that already exist (Vettori, 2018). From a social hermeneutics perspective, the main goal is understanding the building up of realities in particular historical and social settings and how they are exactly formed (Vettori, 2018). Applied to the educational setting, the hermeneutic approach allows for excavating the dynamics of teaching and learning by scrutinizing the instructional context, and the breadth of meanings in which the instructional plans are made to affect the interactants’ perceived reality (Lueger and Vettori, 2014).

At the core of the hermeneutic approach lies the significance of context and interaction. Hermeneutics is interested in the meaning structures that should be interpreted and inferred from the contextual associations through which they are revealed. It is also noteworthy that hermeneutic approaches do not pursue an objective reality or a single right meaning, yet instead critically explore if a particular reconstruction may be considered a relevant and sustainable theme or not, and also if it is capable of holding its status against the other relevant alternatives. That is to say that though the outcome of routine behaviors is contingent on their normality assumption, the hermeneutic approach sees this normality assumption as the beginning point of reflexive and critical investigations. Thus, enquiring questions from diverse points of view turns into a main constituent element of a hermeneutic method (*cf.* Gadamer, 2004). In recent years, L2 scholars interested in the domain of psychology of language learning and teaching have applied some innovative approaches to explore different aspects of psychological factors involved in the process of L2 language learning and teaching (Derakhshan, 2022). However, the use of the approach is still in its fledgling state and it is believed that this method can provide deeper insights into the underpinning factors involved in the emergence of these psychological variables. The purpose of this paper is to review the contribution of a hermeneutic approach, especially the modern hermeneutic approach, to the investigation of psychological variables in second language acquisition (SLA).

### Background of the hermeneutic approach

The hermeneutic approach is among the most popular regularized frameworks in qualitative research, which acts as a reliable methodological foundation and relations to different theoretical conventions, including phenomenology (which emphasizes the association of abstract phenomena and the social context accommodating these phenomena: *cf.* Husserl, 1999), institutionalism (according to which the categorization of behaviors and socially constructed common amounts of knowledge are at the core of social communication) and modern systems theory (in which communication is viewed at the core of all social systems *cf.* Luhmann, 2013). The social hermeneutic approach stemmed from socialization and education research projects, and got very well received in institutional research and organization studies. It later on was further developed in international educational research. As the social hermeneutic approach highlights the reconstruction of the relevant values, norm and logics interaction systems of social structures and processes, it addresses questions that are quite relevant for explaining contemporary developments in educational strategies and policy-making (Lueger and Vettori, 2014).

The social hermeneutic approach helps to understand the growth of controversial issues, socially oriented discourses embedded in socially complicated spheres which include a wide array of participants and various points of view such as an educational setting (Vettori, 2018). It helps to investigate the integration of well-established educational plans in wider historical and social settings (e.g., investigating the informal and formal needs of students with various backgrounds to satisfy while learning something, and unraveling the strategies that result from meeting these needs; or evaluating the prerequisites for change in relation to official educational and academic norms (Rammel and Vettori, 2021). It helps to change the basic values, assumptions and norms which represent themselves in instructional settings, learning designs and technologies. It helps to analyze the association of various educational levels in terms of acquisitional development and cultural adaptation, and explore students’ construction of reality and perception patterns and assess their effect on their relationships and educational plans (Lueger and Vettori, 2014).

### Distinctive features and types of hermeneutic approaches

At first, almost all hermeneutic attempts were based on a feature called the hermeneutic circle, which described the recurrent interaction of the interpreter’s previous estimations about the meaning of a text and his/her refinement and edition throughout the interpretation procedure (Gadamer, 2004). Put it simply, making sense of a text as an entirety is possible just in association with its individual constituent elements and the other way round. Despite the fact that the main criticism of the circularity concept is its inherent metaphoric dubiety (*cf.* Shklar, 2004), two basic principles are established by the hermeneutic circle which still may be considered the main features of social hermeneutics too. The first point is the need for the interpreter to constantly doubt his/her present interpretation condition, and should doubtingly investigate each strand of analysis (Rammel and Vettori, 2021). The second point is that to make sense of the text, one cannot just rely on the text itself. Rather, the interpreter needs to refer to the socio-historical and literary contexts in which the text has been produced. Seen this way, we can see why the hermeneutic approach is of interest to educational and social scientific domains (Vettori, 2018). That is because the hermeneutic approach involves an in-depth context analysis according to the original texts and goes beyond just a simple text analysis.

Accordingly, several types of hermeneutic approaches can be thought of, for instance, the sociology of knowledge hermeneutics (Soeffner, 2004a, 2004b), objective hermeneutics (Oevermann et al., 1979; Lueger and Hoffmeyer-Zlotnik, 1994; Reichertz, 2004), genre analysis (Bergmann and Luckmann, 1995), the documentary method (Bohnsack et al., 2010), and life-world analysis (Honer, 2004). All these varieties are largely in debt to the conventional hermeneutic circle. Yet, each has somehow moved in a different direction away from the fundamental model of interpretation. Modern approaches to social hermeneutics move away from attempts to make sense of a text by fluctuating between the whole text and the different constituent elements it is comprised of (Rammel and Vettori, 2021). The modern varieties typically begin with the deconstruction (another distinctive feature of this approach) of the source text. The purpose of this strategy is the generation of new insights and the reduction of the threat of assigning the new findings altogether to certain fundamental presumptions. The myriads of existing approaches also show that these varieties may not be either standardized or used to address a research problem in a similar fashion (Lueger and Vettori, 2014). With this respect, the hermeneutic approach may be seen as a shared framework of methodology which renders principles and ideas available of how to deal with the meaning aspect of social events systematically (Knassmuller and Vettori, 2009). Furthermore, different varieties of the hermeneutic approach share a number of distinctive features as summarized here.

One distinctive feature is a focus on extensity, with the aim of reconstructing and taking into account all possible interpretations. There is no fixed and prescribed regulation on how to interpret a particular event, and since the overlay of several layers of meaning and connotations imply that arriving at a uniquely true interpretation of a statement is hardly possible, formulating appropriate questions for a text, and assuring not to overlook or exclude any meaning alternative that may be relevant are two of the major prerequisites of an interpretation process guided by the hermeneutic approach. Another distinctive feature is the deconstruction of the source text, which entails breaking it in more manageable parts and setting some criteria for the validity of each strand of analysis to constantly check the interpretations (Oevermann, 2002). Unlike the classic hermeneutics and what was formerly introduced as the hermeneutic circle, the modern hermeneutic varieties refrain from dealing with the text as an entirety, in which every former reading oscillates between particularism and holism. This oscillation gives way to the hypotheses being tested and formulated in each consequent reading. The other distinctive feature is the sequentiality principle, which means perceiving the internal structure of the text as several sequences the particular time order of which is a major cue for the underpinning structures of meaning. This feature relies on the belief that meaning structures also adhere to a sequentiality principle (*cf.* Oevermann, 2002). According to Lueger (2010), just a method that follows this sequentiality principle can interpret how actions and interactions are organized.

### A modern hermeneutic approach to SLA

Whereas classic hermeneutics mostly dealt with interpreting philosophical or literary texts, modern hermeneutics turns the focus of interpretation to the non-verbal and verbal aspects of communication too as presuppositions, pre-understanding and semiotics (Rammel and Vettori, 2021). Modern hermeneutical approach can be coupled with the acquisition of four language skills including reading, writing, listening and speaking, to improve language learners’ comprehensive and interpretative outlooks (Seth, 2020). Language learners can use this approach and incorporate private discourse with the modern hermeneutical method to foster better quality and more effective communication marked by integrated prior knowledge, cognitive and linguistic skills altogether (Seth, 2020).

To discuss how a hermeneutic examination of the language learning process is related to the theory and practice of L2 teaching, one relationship can be thought of as rooted in the fundamental concern of hermeneutics with coming to a clear understanding, as a common purpose of L2 researchers and teachers. As convincingly discussed by Ochsner (1979), hermeneutics can adopt a key role in theory development and research methodology design in the SLA domain. The introduction of diary research as beneficial method of research in SLA confirms the increasing popularity of Ochsner’s argument. In a more well-reputed relevant investigation, Bailey (1983) reckons that awareness of the procedures of both hermeneutics and empirical science can offer researchers all they need to explore the mechanism underlying the SLA accounted for by these two points of view that complement each other. Since a goal of diary works of research is to facilitate a more comprehensive understanding of SLA *via* self-reflection and introspection with the learner being engaged in learning process of L2, Bailey refers to this research method as emergent from the convention of hermeneutic science.

As an example, Murphy (1989) discusses how hermeneutics is related to the listening skill process in L2. In an article that firmly locates the first language speech communication discipline in hermeneutics, Stewart (1983) draws attention to four main ideas at the core of the hermeneutic science of modem times. An awareness of these core ideas can result in a philosophically rich point of view toward the listening skill in language acquisition. The four core ideas included a fusion of horizons, play, linguisticality, and openness. Meanings are reckoned to be open which implies that the interpreter takes part in the generation of interpretations when language is used. When speakers speak, they do not elaborate on the content which is easily directly decoded by listeners. Only during interactions with speakers, listeners are perceived as complicit in meaning production. The messages emerging from speaker-listener interactions are dependent on historical, situational, linguistic and cultural settings.

In a review of the past twenty-five years of teaching second language listening comprehension, Brown (1987) pinpoints that language is entirely derived from, encoded and decoded as contextualized. An emphasis of hermeneutics on openness further proves this idea. It proves that in relation to all kinds of human knowledge (literature, history, science, law, music, etc.), all interpretations are naturally context-dependent and historically oriented. The perceiver cannot be separated from the act of perception. The same goes for the outcome of something perceived. Besides, hermeneutics contends that all human interpretations are inevitably tentative, conditional, and prone to changes in present or future time (Vettori, 2018). What we produce or comprehend (both production-based and comprehension-based language skills) does not result from a closed or fixed system. Rather, it is prone to constant growth and change. Correctness, truthfulness, and accuracy are not what is basically the matter. What actually happens during language-mediated communication is a message co-construction among interlocuters. Speakers and listeners are actively engaged in a collaborative process of meaning-making. In the hermeneutic convention, the perceiver’s subjectivity is viewed as a major dimension of the comprehension process (Murphy, 1989).

### Hermeneutic approach to psychological variables in SLA

SLA research has witnessed a shift in recent years from negative psychology to positive psychology (Mac Intyre and Gregersen, 2012; MacIntyre and Mercer, 2014; Derakhshan, 2022; Wang et al., 2022). Negative psychology deals with excessive concern with the affective filter and the perceived impeding effects of such variables as L2 anxiety on language learning but positive psychology welcomes research on both negative and positive affective factors in the process of L2 learning (Dewaele and Li, 2020). Rich (2017) highlighted the potential benefits of different forms of qualitative research including social hermeneutics to enrich positive psychology, by noting several forms of qualitative research. Gergen et al. (2015) drew attention to three approaches to qualitative research which could provide deeper knowledge than could be provided by the quantitative approach and presented their implications for positive psychology. Among these three approaches to qualitative research was hermeneutics and social understanding.

Four qualitative methods subsumed under the social hermeneutic approach, which could adequately contribute to positive psychology are the auto-ethnography, narrative, performative and phenomenological methods (Creswell, 2009, 2013; Denzin and Lincoln, 2011). Wertz (2015) provided an insightful review of the phenomenological types and their uses in positive psychology. Among famous psychologists who used the phenomenological and narrative types of data in his investigations, occasionally from sources of biography, we can mention James and his original volume entitled as the “Varieties of Religious Experience” (James, 1997). Also, Csikszentmihalyi (1990) used another variety of hermeneutic approaches which led to the production of rich data. This researcher mixed open-ended and semi-structured interviews with data obtained from quantitative surveys, and managed to make more experience-based, context-specific psychological investigations than were possible in standard quantitative methods. Varieties of the hermeneutic approach have the advantage of allowing for realistic and lived-experiential data collection from more naturalistic contexts (Rich, 2017).

Other researchers and especially philosophers, for example, Crease (1997), have combined phenomenological methods and analysis of performance, which adds a further dimension to hermeneutic approaches. Performance approach is a less frequently used variety of hermeneutics in positive psychology (Rammel and Vettori, 2021). One application can be to use public performances in an attempt to enhance associations with the audience or with the realistic experience of the target population which may, otherwise, be embedded in cultural or social worlds differing from the performers (Lueger and Vettori, 2014; Vettori, 2018). However, again at last researchers can effectively mix qualitative methods including performance, with quantitative methods, as probably in evaluation inquiries (e.g., Martens, 2014; Patton, 2015), to find out, for example, whether the audience of a performance about teaching a certain technique (e.g., stress management technique) actually alter their behavior afterwards or not. Some fields of study appear to make better use of these hermeneutic varieties than the SLA domain. For instance, the strengths-based social work movement (Saleeby, 2012) has been historically better receptive of different approaches to methodology and has managed to see practical implications and induce positive social variation (Rich, 2017).

Besides positive psychology that has affected SLA research in recent years (see Dewaele and Li, 2020), the effect of the social constructionist approach is comparatively as strong. The socio-constructivist view of language learning favors qualitative and interpretive research following the hermeneutic approach because of the concern with the social construction of meaning and the underlying mechanisms accounting for this process (Willig, 2019). The second language (L2) researcher or teacher shows interest in how language learners develop certain psychological traits (e.g., growth mindset, L2 grit; see Elahi Shirvan et al., 2021a, 2021b) within the social interactions involved in classroom-based language learning and how these classroom interactions construct specific manifestations of social reality. Students are viewed as actors whose mind and behavior are developed in the social context of classroom learning which is comprised of discourses (i.e., how things are talked about) and social effects (i.e., how things are done) that influence and shape how students develop different positive or negative emotions. More specifically, language learners engage in different social interactions throughout their language learning within the ecology of their classroom. Social constructionist L2 researchers employ different approaches of discourse analysis to interpret how these social interactions affect the emergence of different learner personality traits or affective variables. Hermeneutics interpretive process and qualitative research has, thus, many promises for exploring L2 psychological variables.

### Exemplary works of research

Cilliers and Flotman (2016) investigated the psychological well-being in master’s students. They described this psychological variable as a contributing factor to having a productive, enjoyable, and meaningful experience in student life. The goal of their study was to offer a qualitative account of the psychological well-being levels of junior students affiliated with a part-time master’s degree coursework in Industrial and Organizational Psychology so as to enhance an empathetic interpretation of their experiences. These researchers sought to achieve an understanding of how these master’s students’ experienced psychological well-being could help university the relevant departments at the university to facilitate the proper and adequate psychological support to students and the improvement of their resilience in productively and effectively fulfilling their first-year academic life and maybe their master’s degree as well.

To conduct this study, Cilliers and Flotman (2016) did some qualitative research within a hermeneutic interpretive framework. They collected their data from a focus group of 10 participants selected conveniently. A thematic content analysis was used which led to the extraction of eight themes. These themes were then interpreted in association with the existing psychological well-being literature. The major findings showed that student distress which was induced by job demands resulted in an overwhelmed languishing feeling. To the contrary, student eustress caused by job-related resources could result in a sort of flourishment, together with locus of control, optimism and self-efficacy. As for the pedagogical implications, university industrial and organizational psychology faculties could use the findings for a better and more in-depth understanding of their students’ experiences of psychological health, which can contribute to the students’ timely and successful completion of their education. This research contributes to the literature about master’s students’ actual positive and negative experiences and psychological health that is often neglected by university faculties to be idiosyncratic.

Though the above-mentioned study was not conducted in SLA domain, it can be replicated in L2 studies, as the focus was on using hermeneutics in investigating a psychological factor. The next exemplary work of research has been done among English as a foreign language teaching (EFL) teachers and is, thus, more relevant to the field.

In their research, Ramezanzadeh and Rezaei (2019) focused their research on English language professors in higher education, and their experiences of fostering their learners’ authenticity. Based on Barnett’s (Barnett, 2007) authenticity theory as the perceptual theoretical framework, authenticity was explored as an authentic voice in this research instead of an inherent quality of the materials produced by native speakers. Interviews and personal documents were used for data collection. The interpretation process of modern social science hermeneutics was used for data analysis. Three major themes were found, including critical knowledge in the ELT domain, reflective and dialectical praxis, as well as a localized and flexible curriculum. In fact, the results of this research trespassed the exclusive border of non-native and native L2 speakers. The results also showed that the development of perceived authenticity in English language learners requires a climate receptive of for inclusion and diversity besides ontological, practical and epistemological spaces. Moreover, the research participants showed that authenticity can be grown in their students *via* critical knowledge that may be gained from discourses with not just the majority of voices but also those being marginalized. The empowering implication of these findings point to the promising contributions of the hermeneutic line of inquiry into L2 psychological variables.

Also, very recently, Elahi Shirvan and Taherian (2022) applied the modern hermeneutics approach to explore the actualization process of the potential affordances for foreign language enjoyment (FLE) within the ecology of an L2 classroom. Having collected their first-round interview data from an L2 language teacher, these researchers started their encounter with the transcripts of the interviews to extract the range of thoughts underlying the actualized affordances for FLE. Secondly, they came up with the identification of the main themes regarding the way the FLE affordances are actualized in the ecology of the classroom. Thirdly, they categorized the emerging themes and; finally, illustrated the summary of the established themes.

## Conclusion

To help L2 teachers and researchers who seek to recognize and effectively satisfy the psychological needs of their language learners, the sharp-eyed lenses of hermeneutics to delve into cognitive and affective factors, and the inner speech can allow for a better conceptualization of the underlying processes of how student think and feel in class while engaged in language learning (Murphy, 1989). Hermeneutic methods in investigating psychological variables provide the chances of connecting society and academia more broadly in ways hardly ever seen before with the quantitative approach. These forms of qualitative research may help to bridge the existing gap between psychologists and techniques among community members including performance-based research and action research are exemplary hermeneutic methods that can pull both groups close to each other (Bringle and Duffy, 1998; Schutt, 2014).

Hermeneutic methods, contrary to many quantitative research methods, are also more likely to facilitate efficient communication among the overall public, because the members are barely literate in graduate level quantitative methods or statistics (e.g., Creswell, 2009, 2013). We can expect to predict positive psychologists efficiently share the results of a hermeneutic-guided positive psychology to many audiences, and the findings can have evident implications for social variation, both on the individual scale and on the family scale, and on the sociocultural scale (e.g., Psychology Day at the UN, 2014). Perceiving the benefits of some hermeneutic positive psychology can be translated as hard work. According to Gergen et al. (2015), the power of tradition is not negligible. The structures of the organization are strong. Though the quantitative approach to investigating L2 psychological variables has been dominant, maybe here is a position marked by the relative youth of positive psychology, which can be considered a positive point concerning the potential research approach and the associated shifts of disciplines (e.g., Kuhn, 1962).

What maturing positive psychology has to offer is still being discovered, and maybe a work of research like the present study can act as a turning point in prospective research. L2 psychological investigations can be more than prediction and measurement, and may entail attempts to promote understanding, criticism of formerly presumed truths, and inquiries that can have evident social implications. What the forthcoming decade can be expected to hold for hermeneutic-driven line of inquiry about L2 psychology is still indeterminate, yet there can be hopes that certain major changes are to happen to the realm of research.

### Suggestions for further research

Investigations of L2 psychological variables especially influenced by positive psychology and the socioconstructivist approach in recent years have been mostly quantitative in type. Thus, there seems to be a need for employing qualitative hermeneutic approaches to accumulate and analyze psychological knowledge about language learners. Considering the situated nature of the development of L2 psychological variables, the hermeneutic approach seems to be fit for collecting real-life in-depth data from language learners as they actually experience the process of language learning. There are many positive and negative psychological variables that can be explored in depth through the varieties of hermeneutic approaches (e.g., phenomenology, momentary assessment, etc.). Some of them have been explored more before in the history of psychological L2 studies, such as L2 motivation and anxiety, while some have been only confined to the past few years. Examples are L2 grit, growth mindset, passion for learning and so on. Some have been just borrowed from other domains for the SLA studies, and have not yet been defined domain-specifically (e.g., L2 compassion).

Another point to consider is that more longitudinal studies are needed in SLA research to explore the process of L2 psychology development or co-development throughout an entire course in class-based interpersonal relationships (see Kruk et al., 2022). Changes are not expected to occur out of classroom interactions and overnight. The details of the teacher-student and student–student relationships especially in interactive tasks and activities should be examined to see how they can lead to the formation of certain psychological variables, personality traits, emotions, attitudes and so on (see Xie and Derakhshan, 2021). For sure, such longitudinal studies, which can be qualitative and hermeneutic in type, need innovative research approaches that are compatible with the dynamic approach to language learning. Researchers interested in these innovative approaches are suggested to read Hiver and Al-Hoorie (2019).

## Author contributions

The author confirms being the sole contributor of this work and has approved it for publication.

## Funding

This paper was sponsored by Anshan Normal University, entitled ‘Research on the Cultivation of Cultural Awareness and Intercultural Communicative Competence in Senior High School English Teaching (Grant No.: 2020yjsjuzx06)’ and ‘the Cultivation of English Core Competence for Senior High School Students (Grant No.: sszx04809)’.

## Conflict of interest

The author declares that the research was conducted in the absence of any commercial or financial relationships that could be construed as a potential conflict of interest.

## Publisher’s note

All claims expressed in this article are solely those of the authors and do not necessarily represent those of their affiliated organizations, or those of the publisher, the editors and the reviewers. Any product that may be evaluated in this article, or claim that may be made by its manufacturer, is not guaranteed or endorsed by the publisher.
